# Framework Using Multicriteria Analysis for Evaluating the Risk of Musculoskeletal Disorders

**DOI:** 10.3390/s25020444

**Published:** 2025-01-14

**Authors:** Karolis Senvaitis, Aušra Adomavičienė, Kristina Daunoravičienė

**Affiliations:** 1 Department of Biomechanical Engineering, Vilnius Gediminas Technical University, LT-10223 Vilnius, Lithuania; 2 Department of Rehabilitation, Physical and Sports Medicine, Vilnius University, LT-01131 Vilnius, Lithuania; ausra.adomaviciene@mf.vu.lt

**Keywords:** biomechanics, risk model, statistics, lifting motion, musculoskeletal disorder

## Abstract

This study includes musculoskeletal disorder (MSD) risk evaluation based on the IMU sensor data gathered from patient-lifting movement performed by healthcare specialists. This is a continuation of previous research focusing on a novel multicriteria statistical model integrating experimental and large-scale statistical datasets. The proposed model estimates MSD probabilities over 5, 10, and 15 years for the neck (0.537 ± 0.156), shoulder (0.449 ± 0.084), and elbows (0.277 ± 0.221). The model enables individual risk profiling, influenced by dynamic parameters that can reduce the long-term risk by up to 70.49%. The model is in its early development stages, i.e., it is the proof of concept that offers a new approach to assessing MSD risk at work using motion tracking data in combination with statistics. Further studies with larger sample sizes and validated criterion weights are needed to refine and validate this approach.

## 1. Introduction

Studies of repetitive motions and work ergonomics examine tasks involving repeated movements, which are particularly common in industrial and office environments. The goal of these studies is to identify the harmful movements or their segments and find alternatives that can reduce the risk of acute and long-term injuries and minimize muscle tension and pain, thereby improving occupational safety and employee well-being. The medical sector faces the same risks, being heavily involved in challenges that require handling live patients. This is even harder to define in research and control in practice [1]. People spend the majority of their time in the work environment, so monotonous tasks that cause minor muscle tension can have a significant impact if postures and actions are repeatedly maintained over many years [2]. This can lead to the accumulation of chronic biomechanical disorders, resulting in an inability to function fully at work or in everyday life. However, modeling these conditions often requires handling a lot of limitations and complexity. Research boundary conditions determine the assumptions, complexity, and level of detail used to analyze a given problem. In biomechanics, it is common to analyze a fragment of motion, such as a stroke, cycle, or similar definition, thereby minimizing the amount of data while maintaining the required accuracy or the level of precision dictated by technical capabilities [3].

Patients in healthcare settings deserve high-quality care, which includes safe transfer and transportation [4,5]. However, it is equally crucial to consider the well-being of healthcare professionals aiming to maintain their work capacity and job satisfaction and reduce the risk of becoming patients themselves. Evidence-based practice highlights the importance of ensuring the physical and psychological safety, health, and comfort of healthcare professionals and their assistants through ergonomic work environments, the use of assistive devices and equipment, appropriate workloads, adequate staffing, and other supportive measures [6,7]. Healthcare professionals, especially rehabilitation specialists and nurses, face significant health risks when performing patient transfers, such as lifting patients in and out of beds or wheelchairs. These tasks often involve awkward body postures, leading to improper body mechanics, rapid movements, and sudden transitions from static to dynamic positions, frequently requiring excessive force beyond an individual’s capacity. The varying physical characteristics of patients and the close physical proximity during transfers can result in unbalanced, incorrect, and uncomfortable postures.

Trauma risk modeling techniques can be employed to assess and mitigate these risks more accurately. By developing detailed models of patient transfer tasks, healthcare providers can identify specific risk factors, such as the patient’s weight, the height of the transfer surface, and the caregiver’s strength and flexibility. These models can then be used to simulate different transfer scenarios and evaluate the potential for injury. Additionally, by quantifying biomechanical risks, this model may help identify the root causes of pain in musculoskeletal disorders, enabling targeted prevention strategies and informing pain management protocols during rehabilitation. Similar approaches have been applied in sports contexts, such as evaluating pain and injury risk in surfing practice, where tailored rehabilitation strategies are critical for managing musculoskeletal pain and optimizing recovery [8]. By integrating data from wearable sensors, force plates, and motion capture systems, researchers can develop personalized risk profiles for individual healthcare workers and identify high-risk tasks. To develop such a model for chronic MSDs, continuous study is required. This approach can help identify differences between individuals who have experienced injuries or pain and those who have not. When addressing chronic injuries, it is particularly challenging to isolate the impact of daily life activities. Injuries can occur in various tissues with distinct mechanical properties and functional conditions, such as bones, muscles, tendons, and cartilage. Injuries to these structures are associated with damage to their mechanical properties, often caused by sudden or chronic environmental loads [9]. Although beneficial, a prolonged study was not conducted, and an alternative method for obtaining risk factors is presented. The application of this risk model extends beyond healthcare, providing insights into the prevention of work-related musculoskeletal disorders and sports injuries by identifying high-risk movements and behaviors. Ultimately, the goal of trauma risk modeling is to inform the design of safer patient handling equipment, the development of evidence-based training programs, and the optimization of clinical workflows.

This study combines experimental motion-capture data with statistical analyses to construct a comprehensive multicriteria model for predicting musculoskeletal disorder risks. The approach focuses on three key phases: motion data collection during patient-lifting scenarios, the integration of cumulative force profiles and joint moments, and the extrapolation of risk probabilities using weighted criteria. This novel framework facilitates individual risk profiling and provides insights into long-term occupational health risks for healthcare professionals.

This paper presents research analyzing healthcare specialists during patient lifting motions. This study proposes a multicriteria statistical model that combines individualized motion capture data and broader population statistics to predict long-term MSD risks.

## 2. Materials and Methods

### 2.1. Research Procedure

**Figure 1:**
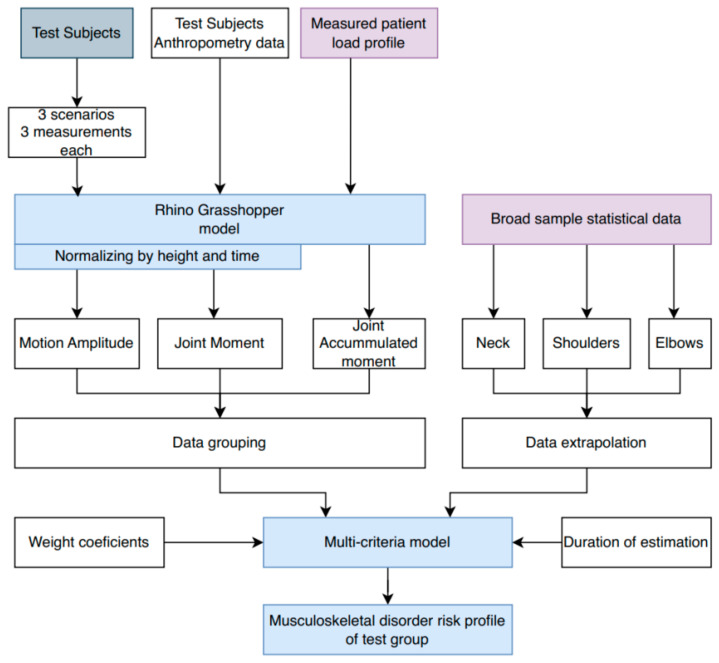
Flow chart of study procedure.

This study followed a structured three-phase research procedure to develop and validate a predictive musculoskeletal disorder (MSD) risk model. First, during the data collection phase, patient-lifting movements were recorded using Xsens Movella IMU sensors in three distinct scenarios: (1) the patient not assisting, (2) the patient assisting by placing their hands on the participant’s shoulders, and (3) the patient not assisting, with the participant wearing an ergonomic belt. During all three scenarios, the patient was holding around 30% of body weight on her own. Each of the 44 participants (16 men and 28 women) performed three trials per scenario, resulting in a total of 396 measurements. The average age of participants was 28 ± 12 years, with a height of 175.11 ± 11.58 cm, and a weight of 75.30 ± 12.58 kg. Supplementary patient load profiles were determined in a separate experiment by estimating reaction forces and residual loads, providing crucial biomechanical parameters for the model. Figure 1 presents the study procedure flowchart, detailing the sequential steps from data collection to risk estimation. Second, during the model development phase, experimental motion capture data were combined with statistical datasets from prior studies to enhance the robustness of the model. Baseline MSD probabilities for the neck, shoulders, and elbows were calculated using statistical regression based on large-scale population data. A multicriteria statistical approach was employed, with parameters such as physical readiness, motion symmetry, and cumulative joint moments assigned weighted coefficients based on their relative importance in predicting MSD risks. The detailed weighting criteria and their application in the model are described further in the methodology section. Finally, in the risk estimation phase, MSD probabilities were extrapolated over 5, 10, and 15 years for each participant, accounting for variations in ergonomic factors and dynamic movements observed during the study. Individualized risk profiles were generated, highlighting the impact of specific factors like physical fitness, technique, and equipment use on long-term MSD risks. By combining experimental sensor data with comprehensive statistical analyses, this procedure established a foundation for evidence-based recommendations to mitigate MSD risks in healthcare professionals.

Cumulative loads, such as the total moment of force, are used as a crucial component for long-term risk extrapolation. It was decided to develop a multicriteria model to predict long-term injury risks. The main drawbacks identified in this model are subjectivity, the need for a significant amount of data, and the necessity to simplify computational conditions. However, the model offers considerable flexibility by allowing updates to individual criteria or related information and prioritizing input data based on its importance.

The model evaluation is based on observable differences between various data segments that influence ergonomic criteria. Due to the weighted criteria and the model’s specificity, parameters can be added or removed, allowing for easy adaptation to changes. This flexibility enables refinement of the model through additional research. The model is based on clear parametric assumptions, components, and their weighted criteria, which are selected based on the most statistically significant groups identified in this study.

The initial weighted criteria are constructed based on the scope of this research, and a prognostic risk assessment is conducted for each participant. One of the most important criteria in the model is the total moment of force in segments, as it directly reflects the load on a joint during movement. Additionally, the cumulative moment allows us to extrapolate the joint load over a defined time or number of repetitions.

A Rhino Grasshopper model is used [10,11] to calculate motion amplitude, joint moment, and accumulated moment. Calculation is performed on the neck, shoulders, and elbows. In parallel, research on MSD is conducted to gather sufficient datasets that would provide a base risk. This base profile is determined by using data extrapolation tools. All these data are incorporated into the multi-criteria statistical model, along with additional inputs such as weight coefficients and duration over which risk is estimated. A more detailed analysis of the data used and methods applied in the model are presented in further research. Once provided with all the necessary data, this multi-criteria model generates an individual risk profile for each test subject across different durations of the estimated timeline.

### 2.2. Research Experimental and Statistical Data

The study integrates experimental patient-lifting data [11] with statistical datasets to develop a prognostic MSD risk model. The first dataset consists of experimental research where patient-lifting motion is being analyzed by equipping test subjects with the Xsens Movella Awinda costume, consisting of standard 17 IMUs (MTw2-3A7G6) [12]. For the research, a mathematical model from the previous research [11] was utilized to process the initial data received from measurements: location of joints at each frame, relative speed, and acceleration of body segments. The start condition for all the different scenarios was the N-pose, involving the test subject standing straight, with arms down. The same condition was used when ending the motion recording. Afterward, the datasets were manually reviewed to consist of distinctive and separate segments for each of the measurements.

**Figure 2:**
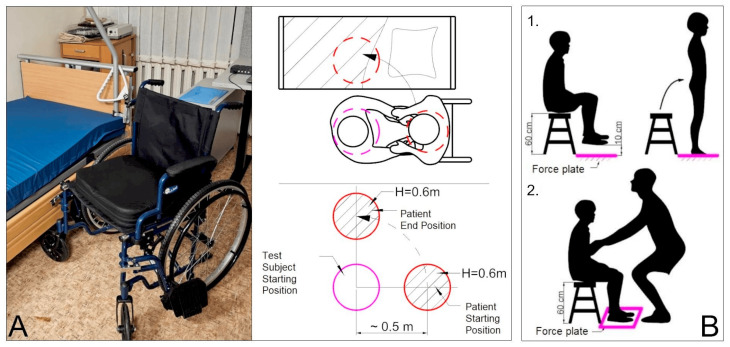
The experiment lifting procedure environment and schematical view: (**A**) patient transfer motion capture setup; (**B**) patient load evaluation: (**B.1**) patient reaction force estimation; and (**B.2**) patient residual reaction force estimation [13].

The analyzed scenarios are evaluated as part of the statistical model. The supplementary measurement is performed to determine the patient load profile, which is then integrated into the mathematical model. Both data-gathering experiments are described in more detail in our previous article [12], while the experiment procedure is presented in Figure 2. The sample size was chosen to have above 0.95 statistical confidence for this cross-sectional study. Experimental research was conducted on a real healthcare specialist and was further supported by the bioethics council permit (2023/3-1504-959).

The second dataset is obtained by analyzing various studies in the literature where a broader sample of statistical data are presented. The baseline scenario probability is determined by analyzing scientific publications to gather more information and establish the frequency of injuries or their symptoms in different groups. From the conducted search, five key studies that examined a large sample of participants and determined probabilities of MSDs in the neck, shoulders, and elbows were identified. A sample of five studies is used to increase the overall data sample size, expand demographic variation, and thereby improve statistical reliability. Table 1 shows the statistical data of the analyzed research of healthcare specialists’ work-related MSD, grouped accordingly to neck, shoulder, and elbow. These data are further processed using mathematical methods. Extrapolation is made to determine the base statistical baseline for MSDs.

Furthermore, each test subject completed a survey, which included criteria important for mathematical calculations and allowing broader data cross-sections, such as sex, height, and weight.

Physical readiness of each of the test subjects was evaluated qualitatively in a score from 1 to 10, based on physical activities frequency, discipline, etc.

**Figure 3:**
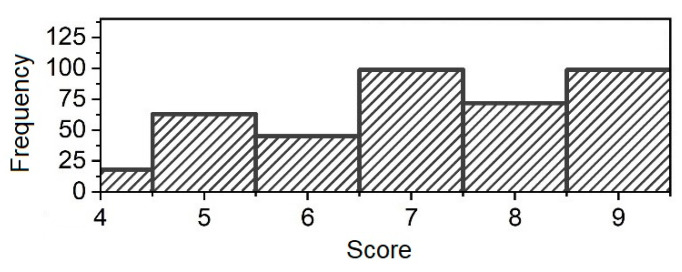
Distribution of test subjects’ physical readiness score.

The scoring evaluation was developed by the team of this research. The distribution of such scores is presented in Figure 3. In terms of physical fitness, the results indicate that there is a relatively wide distribution among fitness levels, allowing the subjects to be classified into low (1–6), medium (7–8), and high (9–10) physical fitness groups.

### 2.3. Matchematical Method

A multicriteria statistical model is developed for the scope of this study. It is assumed that components with higher probability or importance are assigned a greater weighted index. This is called the utility performance index in decision-making processes, where the probability is positively correlated with the above-mentioned coefficients. The mathematical expression of the model is based on published scientific works [14,19]. The model incorporates weighted criteria such as physical readiness, motion symmetry, and cumulative joint moments. Each parameter is assigned a weighting coefficient based on its significance in MSD risk.

The probability of each scenario is derived from the baseline scenario probability, which is based on a larger statistical sample, incorporates a time dimension, and uses actual injury statistics. In this way, the probability of experiencing an injury or symptoms under Scenario A is as follows:

(1) $$p\left(A\right)=Q_{j}\cdot \sum_{i=1}^{n}X_{i},$$

here, *p*(A) represents the probability of scenario A occurring, *Q_j_* is the baseline scenario probability, *X_i_* is the element factor, and *n* is the number of elements considered in the model.

The element factor is a key value in the model with the potential to influence whether the baseline scenario has a higher or lower probability of occurring. The element factor is determined as follows:

(2) $$X_{i}=q_{i}a_{ij},$$

here, *q_i_* is the weight coefficient, and *a_ij_* is the *j*-th likelihood of the *X_i_* element happening at a higher or lower probability.

Element possibilities are defined individually for each data segment, determining the relative weight based on the available data sample. For example, if the average of a metric across the entire research population is Y, the average in the gender-based segment for men is 1.05 Y and for women it is 0.9 Y, then the corresponding *a_ij_* values will be 1.05 and 0.9, assuming a linear relationship. This means that for men there is 5% higher likelihood for event Z to happen, while for women it is 10% lower.

The weighting coefficients are a central metric of the entire model, assigning weights to different scenario possibilities based on their established importance. The weighting coefficient for each scenario is determined as follows:

(3) $$q_{i}=B_{i}{\left(\sum_{i=1}^{n}B_{i}\right)}^{-1},$$

here, *B_i_* is the element weight score, while *n*—number of evaluated elements in the model.

A mathematical condition must be satisfied when defining elements and calculating weighting factors. This condition can also be used to verify whether the weights are correctly distributed:

(4) $$\sum_{i=1}^{n}q_{i}=1,$$

here, *q_i_* is the weight coefficient.

This model allows for the evaluation of both acute and chronic injury risks. The primary limitation of the model is understanding which criteria has greater significance for the probability of the analyzed event. However, this can be assessed through isolated or large-scale studies.

**Figure 4:**
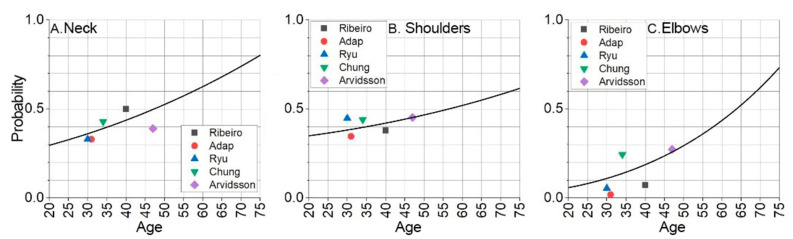
The base probabilities for the neck, shoulder, and elbow to develop musculoskeletal disorder represented with extrapolated curve: (**A**) neck area; (**B**) shoulder area; (**C**) elbow area.

Since this study does not directly investigate injury probability, not all necessary data for risk assessment are derived from the established sample. For this reason, the baseline scenario probability is determined and presented in Table 1. The statistics presented in the table do not directly indicate baseline scenario probabilities; however, by evaluating different scenarios and age conditions, baseline scenario probabilities can be extrapolated. Different sample sizes are considered to have a greater impact on statistical trends. Statistical regression is used to extrapolate curves. The statistical trend is modeled as a quadratic dependence taking the shape of a parabola because, with aging, both the body’s capacity and accumulated load create increasing conditions for the onset of MSD. This approach produces baseline probability curves for each of the analyzed joints (neck, shoulder, and elbow). The extrapolation results for the analyzed age range are shown in Figure 4 and specified in Equations (5)–(7).

The base probability curve for neck musculoskeletal disorder is modeled as follows:

(5) $$y=\left(6.67\cdot 10^{-5}\right)x^{2}+0.003x+0.213.$$

The shoulder musculoskeletal disorder base probability curve is modeled as follows:

(6) $$y=\left(3\cdot 10^{-5}\right)x^{2}+0.0019x+0.3.$$

The elbow musculoskeletal disorder base probability curve is modeled as follows:

(7) $$y=\left(1.767\cdot 10^{-4}\right)x^{2}+0.0047x+0.083.$$

**Figure 5:**
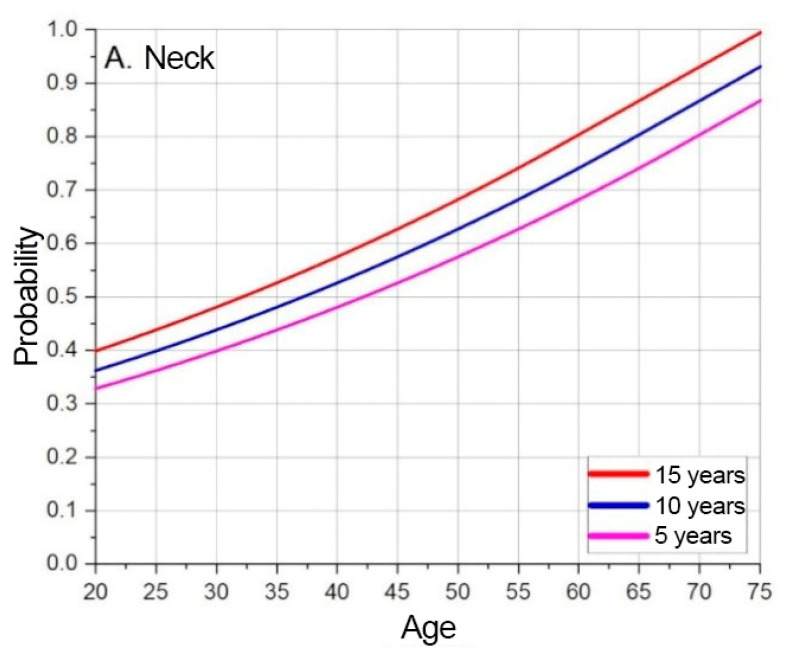
Neck MSD base probability based on predicted timeline.

**Figure 6:**
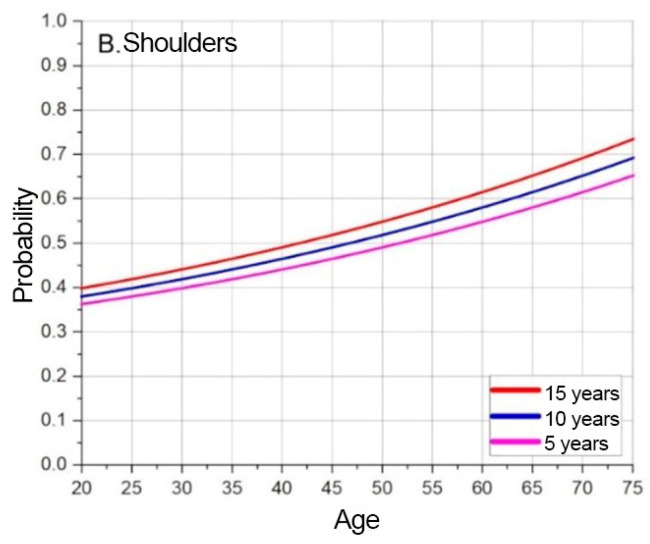
Shoulder MSD base probability based on predicted timeline.

**Figure 7:**
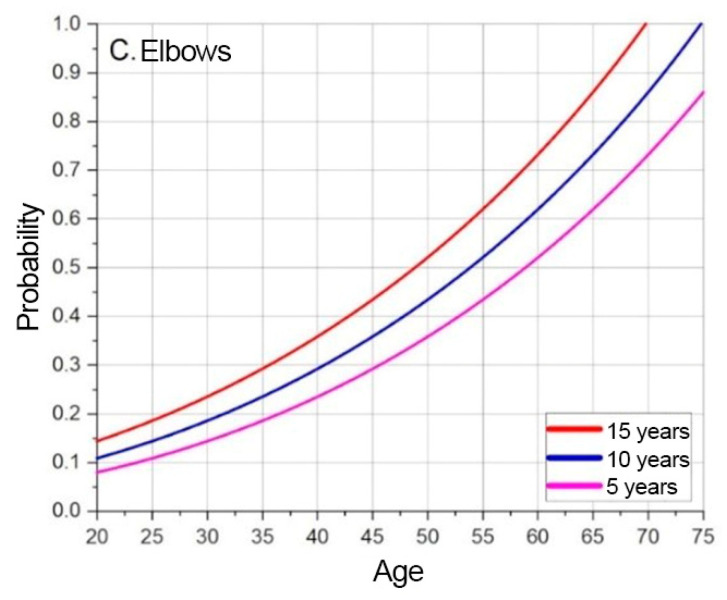
Elbow MSD base probability based on predicted timeline.

The probability of developing a MSD at a given moment, depending on the specified age, is indicated by the extrapolated and offset data. The base extrapolation curves show the risks of developing MSD at the test subject’s current age, at a given moment. The model forecasts MSD probabilities over time by shifting baseline curves. Figure 5, Figure 6 and Figure 7 illustrate these trends for neck, shoulder, and elbow risks. For example, if at age 40 the probability of event A occurring is p(A) = 0.5, and the forecasted scenario is 5 years, then this point is shifted by 5 years. In this way, the probability of event A occurring becomes p(A) = 0.5 at age 35. Using a weighted coefficient criterion, it is then determined whether the probability will increase or decrease under current working conditions and belonging to the current probability group.

Based on the graphs presented in Figure 5, Figure 6 and Figure 7, the baseline probabilities of developing a MSD are determined. Next, by observing trends from the numerical model results, criteria and their weighted coefficients are established to assess whether ergonomic risks will increase or decrease. From the developed risk graphs, the shoulders are in the highest risk zone during the early periods and in the lowest-risk zone over time.

The final undefined component of the risk model is the exact criteria and their corresponding weight values. The assignment of weighted scores is based on three primary data evaluations: range of motion, maximum moment, and cumulative moment. Given that a long-term perspective is being assessed, the cumulative moment is particularly important and is accordingly prioritized in the weighting. The calculation of the criteria’s components involves summing all average deviations from the population’s overall values for each experimental data subset and applying weighting coefficients of 1:1:2 for the range of motion, maximum moment, and cumulative moment, respectively.

Additionally, since discrepancies between moments in the right and left limbs are consistently observed in all measurements, motion symmetry and the relative distribution of movement direction (between sides) are also considered important criteria. Techniques and motion side distributions are relative calculations conducted between two scenarios, predicting the proportional contribution of each element, e.g., 40/60, 30/70, or 50/50. In such cases, the expression applied is the following:

(8) $$a_{ij}^{1}\cdot c_{1}+a_{ij}^{2}\cdot c_{2}=a_{ij},\;when\;c_{1}+c_{2}=1$$

Since defining some elements through absolute numbers or averages results in a loss of precision, elements can also be set parametrically. This allows each subject to provide an individual component value based on variable input data. Parametric criteria are calculated using formulas from the mathematical model and are inputted as datasets rather than single numeric values, as in the case of demographic parameters. In the scope of this research, parametric data—such as range of motion, moment, and cumulative moment—are directly input from the mathematical model into the statistical risk model. This assumes that the individual’s technique remains unchanged throughout the forecast period. It is understood that this assumption is not inherently accurate; however, no data are available on the extent to which the technique may improve. Main model elements with their weighted coefficient and pre-made generalized calculations are shown in Table 2, Table 3 and Table 4, respectively, for the neck, shoulders, and elbows.

It is important to note that the element likelihood (*a*) is a unit that indicates whether the risk of injury for the element is higher or lower. Numbers higher than 1 mean that the chances are increasing, while numbers lower than 1 mean that the chances are decreasing. These sizes were calculated by comparing the sample size of the experimental data of this research to determine the population average and derive likelihoods from that dataset. Using the criteria given in the tables above and research data, the risk for each of the test subjects can be calculated.

## 3. Results

**Figure 8:**
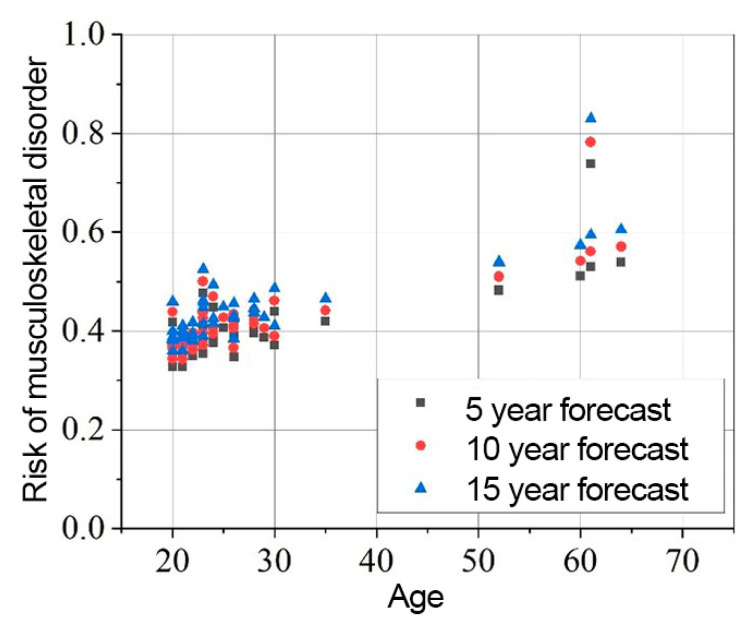
Neck MSD in 5, 10, 15 years statistical probability and age XY graph.

**Figure 9:**
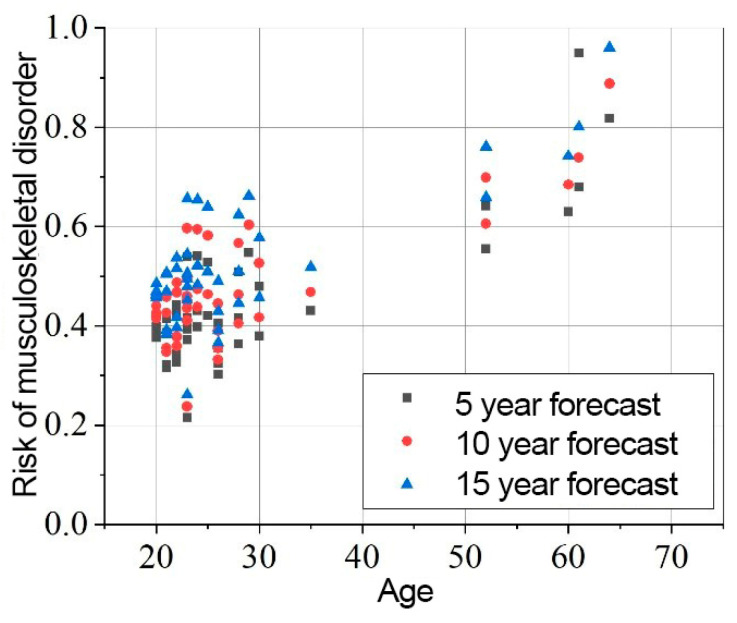
Shoulders MSD in 5, 10, 15 years statistical probability and age XY graph.

**Figure 10:**
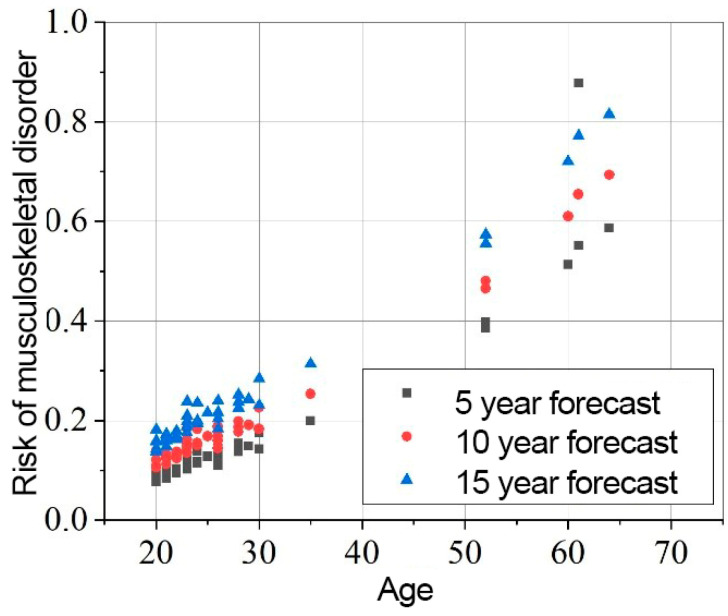
Elbows MSD in 5, 10, 15 years statistical probability and age XY graph.

The results of the risk assessment calculations are shown in Figure 8, Figure 9 and Figure 10. The calculations are performed using the mathematical expression (1), the element factors listed in Table 2, Table 3 and Table 4, the individual anthropometric data of the subjects, and the measurement data obtained during the experiment using IMU sensors. The baseline probability is calculated based on the graphs presented in Figure 5, Figure 6 and Figure 7 (which were generated using the mathematical expressions (5), (6), and (7)).

The results presented in Figure 8, Figure 9 and Figure 10 indicate that the baseline scenario probability is the most critical component of the model. The data distribution, sorted by age, resembles the curves of different scenarios. However, differences between individual subjects are evident, reflecting variations in ergonomics during movement execution. All subjects were trained in safe handling techniques, so no significant differences were expected.

The overall element factor for the neck ranges from 0.614 to 1.542, with an average of 1.119 ± 0.185. The probability of experiencing MSD in the neck region after 5 years is 0.445 ± 0.135, increasing to 0.489 ± 0.145 after 10 years, and 0.537 ± 0.156 after 15 years.

In the shoulder region, the element factor is less variable, ranging from 0.884 to 1.131. The probability of MSD in the shoulders after 5 years is 0.407 ± 0.073, increasing to 0.427 ± 0.078 after 10 years, and 0.449 ± 0.084 after 15 years.

For the elbows, the element factor ranges from 0.916 to 1.587, with an average value of 1.081 ± 0.124. The probability of MSD in the elbows after 5 years is 0.176 ± 0.165, increasing to 0.222 ± 0.191 after 10 years, and 0.277 ± 0.221 after 15 years.

The overall impact of dynamic parameters on long-term risk variability reaches up to 70.49%. When the data are sorted by gender, it is observed that the risk for men is higher than for women. Over a 15-year period, the risk for men is 14.57% higher in the neck, 1.19% higher in the shoulders, and 5.29% higher in the elbows compared to women.

By comparing the model’s outcomes with theoretical extreme data, statistical ranges for specific scenarios can be determined, offering a clearer understanding of the risk domain when modifying different criteria. For example, two scenarios—maximum and minimum—can be compared. The minimum scenario includes criteria associated with the lowest risk, such as optimal technique, good physical fitness, balanced left–right movement distribution, use of ergonomic aids, etc. Conversely, the maximum scenario involves the worst criteria, such as poor technique, etc., with each segment evaluated individually.

Under these conditions, the predicted neck complaint risks for a 20-year-old over a 15-year period range of [0.171:0.529], while shoulder complaint risks range of [0.336:0.440], and elbows complaint risks range of [0.121:0.176]. For a 40-year-old, neck risks are [0.247:0.764], shoulder risks are [0.415:0.544], and elbow risks are [0.315:0.436]. For a 60-year-old, neck risks are [0.346:0.764], shoulder risks are [0.520:0.625], and elbow risks are [0.643:0.811].

It is important to note that this statistical model would benefit from further validation, although the values used in it have been verified in separate studies and have statistically significant relevance. However, the model’s accuracy could be improved by incorporating additional elements, assessing their necessity and importance, refining the examination of relative differences and weighting coefficients, and better substantiating assumptions or determining a more precise baseline scenario. Additionally, it was observed that the cumulative probabilities of the baseline scenario do not follow a linear or other clear trend and may be influenced by demographic differences.

## 4. Discussion

Repetitive tasks in industrial and healthcare environments contribute to acute and chronic injuries, particularly MSDs. In healthcare, lifting patients introduces significant ergonomic risks for professionals, such as nurses and rehabilitation specialists. Current solutions focus on isolated factors but lack comprehensive integration of experimental and statistical data. Our findings of the developed prognostic, multi-criteria risk model provide valuable insights into the factors influencing the likelihood of musculoskeletal complaints over time. The estimated baseline probabilities of neck, shoulder, and elbow disorders reveal a nuanced interaction between occupational demands, physical characteristics, and quality of performed technique. For example, healthcare professionals are particularly susceptible to high short-term loads on their hands and neck [1]. The increased risk observed among male participants, who showed a 14.57% higher probability of neck disorders compared to females, is primarily associated with the larger range of motion used in performing tasks, possibly related to greater differences in physical strength or height.

Dynamic parameters of technique, influencing up to 70.49% of long-term risk variability, highlight the importance of prioritizing ergonomically sound practices, particularly during unavoidable unsafe transfers. While the model’s baseline probabilities are based on large-scale statistical studies, the parametric variations drawn from the experimental data highlight the role of individualized factors like physical fitness, technique, and symmetry in shaping risk profiles. These results emphasize the need for targeted interventions, such as enhanced training and ergonomics optimization, to mitigate risks. For instance, the risk model can help anticipate conditions like cervical radiculopathy in desk workers or shoulder impingements in overhead athletes, enabling early intervention and tailored rehabilitation approaches. Rehabilitation strategies tailored to address musculoskeletal pain have proven effective in sports settings, such as surfing, where personalized interventions are critical for mitigating pain and improving performance [8]. This highlights the potential for integrating such targeted approaches into occupational settings to better manage pain and enhance recovery outcomes. The data suggest that interventions should receive increasing attention as MSDs rise due to our evolving lifestyle [20]. The findings of this study can be integrated into prevention strategies by designing targeted training programs that improve movement ergonomics, particularly in healthcare professionals and athletes prone to neck and shoulder injuries.

However, this is the early phase of the model, and it requires further research and improvements before it can be applied more broadly. Key limitations were identified during the course of this research.

First, the proposed statistical model has not been validated at the current time. Further detailed studies may be needed to validate it as a fully developed tool. Although, it is still difficult to validate a statistical model since probability cannot be quantified and defined as the certainty of an event. However, in the absence of data on the real system, assumptions can be made based on sensitivity analysis [21]. Furthermore, the current weighting criteria have more of a demonstrative purpose. The basis of the weighting is subjective and requires additional and detailed studies to be carried out for each weighting criterion. Although, this model provides great flexibility since each of the criteria can be modified, analyzed, or implemented as a separate fragment of the model, without compromising the functionality of the model.

The element factor is supported by the sample size of this precise study. Although its size is 396 measurements, this is still a relatively limited population of 44 healthcare professionals. This increases the statistical variance when it comes to constructing element factors and determining the probability of the risk that is the influence of various criteria. However, if the model were to be expanded and used more widely, a public database would allow each study to contribute to the study data, increasing the reliability and probability of the element factor variance. This would significantly reduce uncertainty and could help analyze, define, or address various health-related symptoms of MSDs [22].

It is necessary to mention the high complexity of the movements. When a person performs a difficult movement while recording, uncertainty is inevitable. In addition, this study uses a live participant imitating the patient. This expands the area of uncertainty, this being the repeatability of movements, the variability of circumstances from person to person, etc. Also, healthcare professionals often try to help the patient, ignoring the safety instructions presented to them during the participation in the study [4].

Like any other ergonomics-related measurement, this one is no exception. This study estimates risk prediction only in terms of the work environment, although there is a large influence of life outside work. Basic statistics, which use large sample data, help to mitigate this limitation, because real CRS data include other life factors, although in this case, demographic differences between countries, education, and quality of life also have a difference that cannot be ignored.

Although the model has its limitations, it clearly demonstrates the benefits of combining extensive statistics and experimental sensor data. This can be adapted and used in any other field of science or engineering to model various factors. Using it in a narrower engineering setting can lead to even fewer limitations and a more accurate relationship between each element and its weighting factors. The main difference between this study and other works examining MSD that have been reviewed is that none of the models adjust their experimental data with statistics collected from other studies. Many models and risk studies separate their conclusions from the study’s background [23,24,25]. Most other studies perform risk assessments using a questionnaire or modeling methods for basic resources. Since this study already incorporated several studies reporting MSD data, the results were not compared with the existing literature. Instead, this study’s results serve as a derivative, indicating increased or decreased risk based on those studies, with the distribution of the base data outlined in the methods section.

Our future work is directed toward obtaining more time-distributed data samples. The sensor measurement experiment is a single point in time when a technique is assessed. The aim is to conduct multiple assessments and collect data on how a technique evolves. Obviously, techniques can change for the better or for the worse, but the influencing factors may not be so clear. A separate study needs to be conducted, aiming to identify and define the factors that would allow us to determine, using only a single measurement, whether a person is likely to deteriorate or improve their technique. This would be useful not only for the risk assessment model but also for all science branches modeling the musculoskeletal system from IMU data, as it would be easily applicable for prevention. The ability to quickly identify risks enables us to prioritize and focus more attention on professionals at higher risk of injury, especially as we work and live in environments with limited resources [3]. Future developments could expand the framework by incorporating rehabilitation techniques that have shown efficacy in sports-related pain management [8], bridging the gap between predictive models and actionable clinical applications.

## 5. Conclusions

This study emphasizes the use of experimental IMU sensor data to broaden the risk base for MSDs. A research model is presented that demonstrates the general applicability of both experimental and broader statistical data by combining them through a multicriteria statistical model. The initial conditions of the model are based on large-scale statistical research, and the parameter fluctuations are derived from data collected during the patient-lifting experimental study. It was found that for the sample group of this study, using the developed statistical model and their performed methodologies, the probability of neck disorders after 15 years is 0.537 ± 0.156, the probability of shoulder disorders—0.449 ± 0.084, and the probability of elbow disorders—0.277 ± 0.21. The main risk factors are high, albeit short-term, loads on the hands and neck and the technique used.

It was found that men are more likely to experience MSDs. It is predicted that over the next 15 years, men working in the health and social care sectors will experience 14.57% more neck, 1.19% more shoulder, and 5.29% more elbow disorders than women. The main factor increasing this risk is the range of motion used by men when performing handling movements. A higher range of motion may be associated with greater differences in physical strength and/or height.

It has been found that the dynamic parameters of the methods performed can influence up to 70.49% of the variable component in the long-term risk calculation. For this reason, to reduce the likelihood of chronic injuries, pain, or other complaints, it is recommended to focus on proper techniques and prioritize them when performing ergonomically unsafe handling movements.

## Figures and Tables

**Table 1:** Research data on the probability of musculoskeletal disorders [14,15,16,17,18].

Reference	Ribeiro et al., 2016 [14]	Adap et al., 2017 [15]	Ryu et al., 2014 [16]	Chung et al., 2013 [17]	Arvidsson et al., 2016 [18]
Research Country	Portugal	India	USA	Taiwan	Sweden
Sample Size	409	212	531	1914	925
Sample average age (±SD)	40 ± 9	31 ± 6	30 ± 7	34 ± 8	47 ± 10
Base risk of musculoskeletal disorder					
Neck	0.501	0.331	0.333	0.434	0.390
Shoulders	0.378	0.346	0.448	0.440	0.453
Elbows	0.072	0.019	0.055	0.245	0.273

**Table 2:** Statistical values of neck multicriteria MSD prognosis values.

Criteria	Physical Readiness			Sex		Technique Scenario		Amplitude	Moment	Cumulative Moment
Weight score (*B*)	1			1		1		1	1	2
Weight coefficient (*q*)	0.143			0.143		0.143		0.143	0.143	0.285
Element title	Low	Medium	High	Male	Female	w/o Belt	w Belt	—	—	—
Element likelihood (*a*)	1.018	0.901	0.161	1.120	0.930	0.952	1.024	—	—	—
Element factor (*X*)	0.146	0.129	0.023	0.160	0.133	0.136	0.146	*a · q*	*a · q*	*a · q*

**Table 3:** Statistical values of shoulder multicriteria MSD prognosis values [made by author].

Criteria	Physical Readiness			Sex		Technique Scenario		Symmetry		Amplitude	Moment	Cumulative Moment
Weight score (*B*)	1			1		1		2		1	1	2
Weight coefficient (*q*)	0.111			0.111		0.111		0.222		0.111	0.111	0.223
Element title	Low	Medium	High	Male	Female	w/o Belt	w Belt	Left	Right	—	—	—
Element likelihood (*a*)	1.009	0.997	1.030	1.016	0.991	0.919	1.041	1.009	0.998	—	—	—
Element factor (*X*)	0.112	0.110	0.114	0.113	0.110	0.102	0.116	0.224	0.222	*a · q*	*a · q*	*a · q*

**Table 4:** Statistical values of elbow multicriteria MSD prognosis values [made by author].

Criteria	Physical Readiness			Sex		Technique Scenario		Symmetry		Amplitude	Moment	Cumulative Moment
Weight score (*B*)	1			1		1		2		1	1	2
Weight coefficient (*q*)	0.111			0.111		0.111		0.222		0.111	0.111	0.222
Element title	Low	Medium	High	Male	Female	w/o Belt	w Belt	Left	Right	—	—	—
Element likelihood (*a*)	1.008	0.998	1.029	1.019	0.989	0.976	1.017	1.005	0.999	—	—	—
Element factor (*X*)	0.112	0.109	0.114	0.114	0.110	0.108	0.113	0.223	0.222	*a · q*	*a · q*	*a · q*

## Data Availability

The statistical dataset with main test subjects’ data has been provided supplementing this research.
